# A Novel Anti-Histone H1 Monoclonal Antibody, SSV Monoclonal Antibody, Improves Lung Injury and Survival in a Mouse Model of Lipopolysaccharide-Induced Sepsis-Like Syndrome

**DOI:** 10.1155/2015/491649

**Published:** 2015-01-11

**Authors:** Toru Kusano, Kuei-Chen Chiang, Masafumi Inomata, Yayoi Shimada, Naoya Ohmori, Takeshi Goto, Shuji Sato, Shigeru Goto, Toshiaki Nakano, Seiji Kawamoto, Yuki Takaoka, Norio Shiraishi, Takayuki Noguchi, Seigo Kitano

**Affiliations:** ^1^Department of Gastroenterological and Pediatric Surgery, Oita University Faculty of Medicine, 1-1 Hasama-machi, Yufu, Oita 879-5593, Japan; ^2^Kazusa Institute for Drug Discovery, Josai International University, Chiba 283-8555, Japan; ^3^Iwao Hospital, Yufuin 879-5102, Japan; ^4^Liver Transplantation Program, Kaohsiung Chang Gung Memorial Hospital, Chang Gung University College of Medicine, Kaohsiung 833, Taiwan; ^5^Department of Molecular Biotechnology, Graduate School of Advanced Science of Matter, Hiroshima University, Higashi-Hiroshima 739-8511, Japan; ^6^Center for Community Medicine, Oita University Faculty of Medicine, Oita 879-5593, Japan; ^7^Anesthesiology and Intensive Care Medicine, Oita University Faculty of Medicine, Oita 879-5593, Japan; ^8^Oita University, Oita 870-1192, Japan

## Abstract

*Background*. Histones play important roles in both host defenses and inflammation related to microbial infection. A peptide mimotope (SSV) was identified from a novel histone H1 monoclonal antibody (16G9 mAb) that was shown to inhibit the mixed lymphocyte reaction. In the present study, an anti-SSV producing hybridoma was established. We investigated the effects of SSV mAb in a mouse acute inflammation model induced by intraperitoneal injection of lipopolysaccharide (LPS). *Methods*. SSV mAb was generated and characterized. Mice were treated with SSV mAb or a control IgG antibody prior to LPS injection. Evaluation of survival rate and lung tissue on histological score was performed. The levels of inflammatory cytokines and histones H1, H3, and H4 in plasma and lung tissue were measured by ELISA. *Results*. Competitive ELISA revealed that SSV mAb binds to histone H1. SSV mAb improved lung injury and prolonged the survival of LPS-injected mice. Increased levels of histones H1, H3, and H4 and inflammatory cytokines (TNF-*α*, IL-1*β*, and IL-6) in plasma and lung tissue after LPS injection were ameliorated by SSV mAb. *Conclusion*. SSV mAb is shown to have anti-inflammatory activity and organ-protective effects, highlighting the importance of controlling histone H1 as well as H3 and H4 levels during inflammation.

## 1. Introduction

Extracellular histones cause various vital reactions during inflammation [1]. Inflammatory stimuli induce deimination of histones in neutrophils. These histones, in complex structures of DNA and granule proteins called nucleosome extracellular traps (NETs), can be secreted into the extracellular space, where they contribute to host defenses as an important bactericidal substance [2, 3]. Recently, it was reported that extracellular histones are elevated in response to traumatic injury and correlate with fibrinolysis and activation of anticoagulants. An increase in histone levels from admission to 6 hr is predictive of mortality, representing evidence of ongoing release of intracellular antigens similar to that seen in sepsis [4]. Extracellular histones, particularly histones H3 and H4, have also been described as mediators of cell damage and organ dysfunction that lead to death in sepsis [1]. Activated protein C (APC) is capable of cleaving and inactivating histones H3 and H4, and coadministration of APC and anti-histone H4 antibody reduces both the cytotoxicity of histones and their contribution to host defenses [1]. Unfortunately, the large-scale clinical study PROWESS-SHOCK revealed that recombinant human APC lacked a significant therapeutic effect for sepsis [5].

In a rat tolerogenic model of orthotopic liver transplantation (OLT), PVG rats that received a liver graft from DA rats survived without immunosuppression [6–8]. We reported that the anti-histone H1 autoantibody is one of the main immunosuppressive factors in plasma induced after OLT in a rat tolerogenic model [9]. Anti-histone H1 polyclonal antibodies reduce the cytotoxic effects of natural killer cells and lymphokine-activated killer cells, induce CD4+ CD25+ regulatory T cells, and promote the differentiation of dendritic cells to a more tolerogenic state [10]. A novel anti-histone H1 IgM monoclonal antibody (16G9 mAb) that was generated to further investigate the mechanism of immunosuppression was shown to inhibit the mixed lymphocyte reaction [11]. 16G9 mAb was found to negatively regulate T cell activation via Treg cell-dependent and Treg cell-independent mechanisms. The results revealed an unexpected regulatory role for T cells for anti-histone H1 antibodies, the overproduction of which is generally considered to be pathogenic in autoimmune settings [12]. A peptide mimotope identified from 16G9 mAb, designated SSV, was shown to be a functional histone H1-binding epitope for 16G9 mAb. Serum antibodies induced by SSV immunization of mice inhibited Con A or anti-CD3-stimulated splenocyte proliferation. SSV immunization in rats before heterotopic heart transplantation resulted in significant prolongation of allograft survival [13].

Previous studies have clinically and experimentally identified bacterial infections as a possible trigger for allograft rejection [14]. We hypothesized that the immunosuppressive activities of 16G9 mAb may be related to the suppression of inflammation. However, 16G9 mAb has a low recovery rate and is of IgM isotype which is predicted to have a lower affinity for histone H1 [11]. To strengthen the affinity of 16G9 mAb, in the present study, we established a hybridoma and purified a mAb against mimotope SSV (SSV mAb) and confirmed that SSV mAb bound to histone H1. Here, inflammatory response was induced by intraperitoneal injection of lipopolysaccharide (LPS) to mice. LPS is a good model to induce the key points of the systemic inflammatory response observed in sepsis. Therefore, this study aimed to investigate the effects of SSV mAb and the importance of controlling histone H1 in a systemic inflammation.

## 2. Materials and Methods

### 2.1. SSV mAb Production

We established a hybridoma that produces SSV mAb. The isotype of the hybridoma was determined using a mouse monoclonal antibody isotyping kit (Sigma, St. Louis, MO, USA). The hybridoma was first cultured in RPMI 1640 medium (Invitrogen Corporation, Carlsbad, CA, USA) containing 20% FBS (Sigma) and antibiotic-antimycotic agents (Invitrogen). Once growth stabilized, large-scale production of antibody was cultured in RPMI 1640 medium containing 2% FBS. The hybridoma was cultured for 1 week, and the culture supernatant was harvested. The protein in the culture supernatant was salted out with 40% ammonium sulfate. The precipitate was dissolved in phosphate-buffered saline (PBS) and dialyzed against PBS overnight. SSV mAb was purified from this solution using a HiTrap NHS-activated column (GE Healthcare Bio-Sciences AB, Uppsala, Sweden) coupled with the peptide SSV conjugated to keyhole limpet hemocyanin (KLH).

### 2.2. Characterization of SSV mAb

The binding of SSV mAb to peptide SSV or histone H1 (Calbiochem, San Diego, CA, USA) was determined by ELISA. In brief, 96-well microtiter plates (Nalgene Nunc International, Roskilde, Denmark) were coated with KLH-conjugated SSV, histone H1, or bovine plasma albumin as control (Wako Pure Chemical Industries, Inc., Osaka, Japan) in 100 mM sodium bicarbonate buffer, pH 9.3. The plates were washed with PBS-Tween 20 (0.05%) and blocked with 3% skim milk and 1% BSA in PBS for 1 hr. Increasing SSV mAb concentrations were added to the wells and incubated for 1 hr. After removing the supernatant and washing, peroxidase anti-mouse IgG1 antibody (Sigma) was added to each well and incubated for 1 hr. After removing this supernatant and washing, the bound SSV mAb was detected using 2,2′-azino-bis(3-ethylbenzothiazoline-6-sulfonic acid) (ABTS) substrate solution (Sigma). A Multiskan Ascent microplate reader (Thermo Fisher Scientific Inc., Waltham, MA, USA) was used to determine the absorbance at 405 nm. The inhibition of binding of SSV mAb to SSV by histones H1, H3, or H4 (Millipore, Billerica, CA, USA) in solution was determined by ELISA. In brief, 96-well microtiter plates were coated with KLH-conjugated SSV peptide (25 ng/well). SSV mAb (10 *μ*g/mL) was preincubated with increasing amounts (0–10 *μ*g/mL) of histones H1, H3, or H4. After 30 min, the mixture was added to the blocked wells coated with SSV. The bound SSV mAb was detected using peroxidase anti-mouse IgG1 antibody (Sigma) as the secondary antibody and ABTS substrate solution for color development. Complementarity determining regions (CDRs) of SSV mAb were identified. Total RNA was isolated from 1.6 × 10^7^ SSV hybridoma cells using the FastPure RNA kit (Takara Bio Inc., Shiga, Japan), according to the manufacturer's instruction. The cDNA needed for RACE PCR was prepared using the SMARTer RACE cDNA Amplification Kit (Clontech Laboratories, Inc., Mountain View, CA, USA). Mouse heavy chain variable region genes (VH) and light chain variable region genes (VL) were amplified by 5′-RACE PCR. The PCR product was cloned into pGEM-T Easy Vector (Promega, Madison, WI, USA), individual clones were sequenced, and CDR was identified using the Kabat database.

### 2.3. In Vivo Evaluation of SSV mAb

Seven-week-old male BALB/c mice weighing 24–26 g (Kyudo, Saga, Japan) were used. The mice were maintained at 25°C with a 12-hour light/dark cycle and given free access to water and standard laboratory chow. The experimental protocol was approved by the Animal Ethics Review Committee of Oita University, Faculty of Medicine. An inflammatory mouse model was induced by intraperitoneal (i.p.) injection of LPS (40 mg/kg), a dose that resulted in 80% of death within 24 hr. The mice were randomly divided into two groups: (1) one injected (i.p.) with anti-mouse IgG (H+L) antibody (IBL, Co., Ltd., USA) as a control group and (2) one injected (i.p.) with SSV mAb as a SSV mAb group. The mice were injected with SSV mAb (4 mg/kg) or anti-mouse IgG antibody (4 mg/kg) 30 min before LPS injection. The mice were treated a second time with the same dose of SSV mAb or anti-mouse IgG antibody 6 hr later. Cumulative survival of both control group and SSV mAb group was evaluated at 24 hr (*n* = 10 each). We monitored the condition of the mice every two hours and recorded the mice which were humanely euthanized with ketamine/xylazine anesthesia when they met certain clinical criteria such as agonal breathing.

### 2.4. Sample Collection

After ketamine/xylazine anesthesia, abdominal cavity and chest were opened and blood sample was collected from the right ventricle. Blood and lung tissue samples of both groups were obtained 0, 3, 6, 9, 12, and 24 hr after LPS injection. Ten mice per group were used at each time point. At each time point, the mice were sacrificed humanely according to the Institutional Animal Care Guidelines of Oita University. Blood sample mixed with heparin was transferred to Eppendorf tubes for centrifugation at 14,000 rpm for 10 min. Prior to harvesting lung tissue, the remaining nonadherent intravascular blood was removed by perfusing the mice with at least 10 mL of 0.9% NaCl by inserting a needle into the beating heart. Aliquots of the plasma and lung tissue samples were stored at −80°C until analysis [15]. To determine the protein content of the lung tissue, samples were weighted, thawed, and homogenized in phosphate-buffered saline and centrifuged at 14,000 rpm for 10 min. Soluble protein concentrations were determined using the DC Protein Assay Reagent (Bio-Rad Laboratories, Hercules, CA, USA). Absorption was measured at 450 nm using a microplate reader (Bio-Rad Laboratories).

### 2.5. Analysis of Histones H1, H3, and H4 Levels in Plasma and Lung Tissue

Plasma and lung tissue histone H1 levels were determined as follows: first, a 96-well microtiter plate was coated with 0.1 *μ*g of anti-histone H1 polyclonal antibody (Santa Cruz Biotechnology Inc., Dallas, TX, USA) in 100 mM sodium bicarbonate buffer (pH 9.3) by overnight incubation at 4°C. The plate was then blocked with SuperBlock T20 (PBS) Blocking Buffer (Thermo Fisher Scientific Inc., Rockford, IL, USA), and plasma and lung tissue samples were added to the wells. Calf thymus histone H1 (Millipore, Billerica, MA, USA) was used as a standard. The mixture was incubated at room temperature for 1 hr. Next, anti-histone H1 monoclonal antibody (Abcam, Cambridge, MA, USA) was added, and the mixture was incubated at room temperature for 1 hr. Peroxidase-conjugated anti-mouse IgG (Santa Cruz Biotechnology Inc.) was then added, and the mixture was incubated at room temperature for 1 hr, followed by the addition of 1-Step Ultra TMB substrate solution (Thermo Fisher Scientific Inc.). Finally, absorption at 450 nm was read using a microplate reader (Bio-Rad Laboratories, Hercules, CA, USA) [16]. Histones H3 and H4 levels in plasma and lung tissue were measured using ELISA kits (Active Motif Inc.; Uscn Life Science Inc.) according to the manufacturer's instructions. Absorption was read at 450 nm, with an optional reference wavelength of 655 nm.

### 2.6. Histopathological Analysis

Lung tissue specimens were inflated and fixed in 10% formaldehyde for histopathological examination. After a paraffin blocking procedure, cross-sections were stained with hematoxylin-eosin. The extent of lung injury was evaluated histologically in a blinded manner, according to the Murakami technique [17]. Twenty-four areas of the lung parenchyma were allotted separate scores for congestion, edema, inflammation, and hemorrhage using a scale from 0 to 4 (0, absent and appearing to be normal; 1, light; 2, moderate; 3, strong; and 4, intense).

### 2.7. Analysis of TNF-*α*, Interleukin- (IL-) 1*β*, IL-6, and IL-10 Levels in Plasma

Plasma TNF-*α*, IL-1*β*, and IL-10 levels were determined using the Bio-Plex Suspension Array System (Bio-Rad Laboratories), according to the manufacturer's instructions. Plasma IL-6 levels were determined by ELISA (Invitrogen Corporation), according to the manufacturer's instructions. Absorption at 450 nm was read on the microplate reader (Bio-Rad Laboratories).

### 2.8. Statistical Analysis

Data are expressed as mean ± standard deviation (SD). Single comparisons were tested for significance using the unpaired* t*-test and Mann-Whitney *U* test. Group pairs were assessed by the Fisher protected least significant difference test. Cumulative probability of overall survival (OS) was estimated by Kaplan-Meier survival methods, and differences between subgroups were assessed by the log-rank test. Differences resulting in a *P* value of <0.05 were considered to be statistically significant for all analyses. All statistical analyses were performed using SPSS 11.0 statistics software (Chicago, IL, USA).

## 3. Results

### 3.1. Characterization of SSV mAb

**Figure 1:**
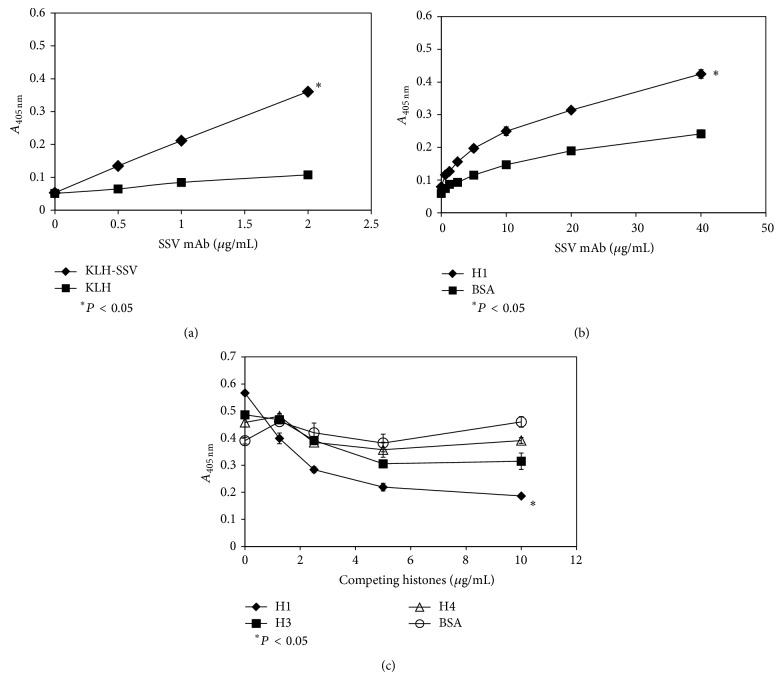
Characterization of SSV mAb. (a) The binding of SSV mAb to KLH-conjugated SSV peptide (KLH-SSV) was determined by ELISA. Native KLH (KLH) was used as a control to eliminate nonspecific binding. Increasing SSV mAb concentrations were added to the wells of a microtiter plate that had been coated with native KLH or KLH-conjugated SSV. After washing and binding of an HRP-conjugated secondary antibody, the amount of SSV mAb was determined by a colorimetric HRP activity assay at 405 nm. (b) SSV mAb binds to histone H1. Increasing SSV mAb concentrations were added to the wells of a microtiter plate that had been coated with histone H1 or BSA as control. After washing and binding of an HRP-conjugated secondary antibody, the amount of SSV mAb was determined as described above. (c) SSV mAb binding to KLH-conjugated SSV was significantly inhibited (*P* < 0.05) by histone H1. In these assays, KLH-conjugated SSV (c) was used to coat the wells of a microtiter plate. SSV mAb was incubated with increasing concentrations of the histones shown in the respective panels, and the SSV mAb-histone mixtures were added to the coated wells blocked with the blocking solution. After washing, the wells were treated with secondary antibody, and the amount of bound SSV mAb in each well was determined as described above. ^*^Statistically significant difference (*P* < 0.05).

We cultured a SSV mAb-producing hybridoma, collected the supernatant after 7 days of culture, and purified SSV mAb using affinity chromatography. The purified SSV mAb was shown to be of the IgG1 isotype, and its purity was demonstrated using SDS-PAGE (data not shown). SSV mAb bound to KLH-conjugated SSV in a dose-dependent manner, and KLH was used as a control to eliminate the possibility of nonspecific binding of SSV mAb to KLH (Figure 1(a)). The binding of SSV mAb to immobilized histone H1 was investigated by ELISA. SSV mAb bound to histone H1 dose dependently (Figure 1(b)), as well as to histone H3 or H4 (data not shown). Since the conformation of the antigen may be different when it is fixed on a plate or in solution, we performed competitive ELISA to confirm the binding of SSV mAb to histone H1 in solution. Histone H1 was a potent inhibitor of the binding of SSV mAb to SSV compared with histones H3 and H4 (Figure 1(c)). The complementarity determining regions (CDRs) of SSV mAb were identified as described in the Materials and Methods section (Table 1). The CDRs were identified for the future humanization of SSV mAb.

### 3.2. SSV mAb Improves Mouse Survival

**Figure 2:**
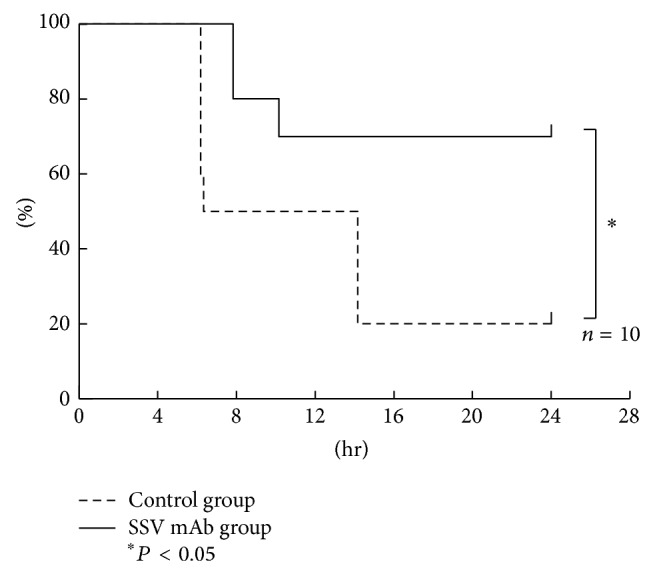
Survival of mice after LPS injection. Survival rates of mice injected intraperitoneally with LPS while being treated with SSV mAb or control antibody. Mice (*n* = 10) were treated with SSV antibody (4 mg/kg) or anti-mouse IgG antibody (4 mg/kg; the control group) 30 min before receiving LPS injection (i.p.; 40 mg/kg). A second, identical antibody treatment was administered 6 hr after LPS injection. The survival rate (%) is plotted versus time (h) after LPS injection. The survival rate of the SSV mAb group was significantly improved compared with the control group (*P* < 0.05) 24 hr after LPS injection.

A LPS-induced mouse model was used to evaluate the anti-inflammatory effect of SSV mAb. Mice were treated with SSV mAb or a control anti-mouse IgG antibody 30 min prior to and 6 hr after LPS injection and then observed for 24 hr after treatment. The SSV mAb-treated mice experienced a 70% survival rate, which was significantly improved compared with that of the control mice (Figure 2) (20%; *P* < 0.05).

### 3.3. Histones H1, H3, and H4 Levels in Plasma and Lung Tissue

**Figure 3:**
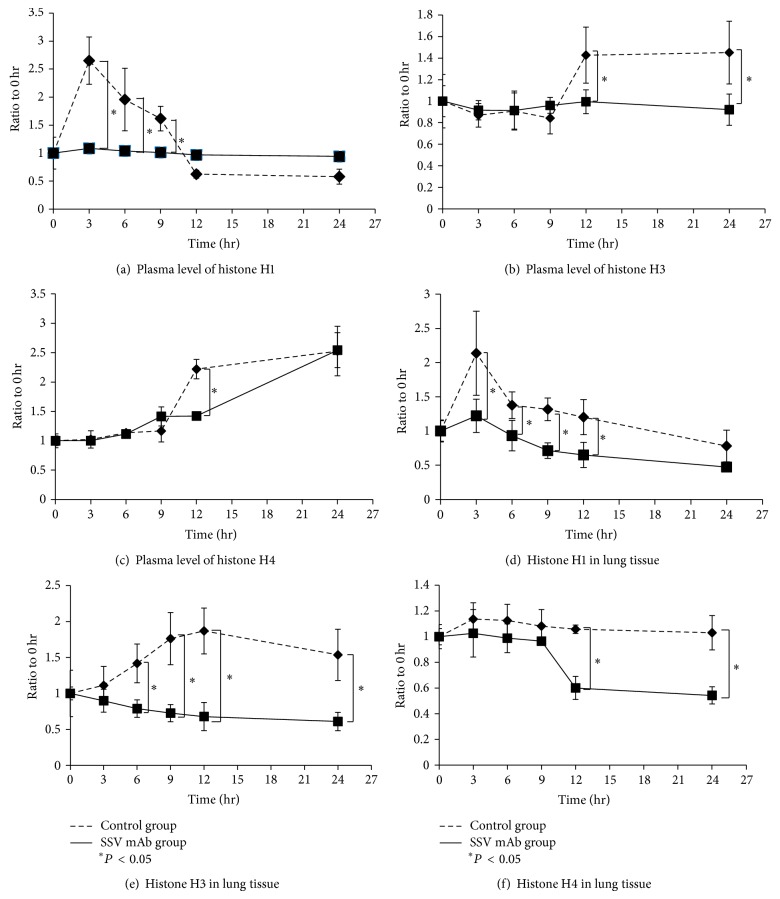
Histones H1, H3, and H4 levels in plasma and lung tissue. (a) Plasma histone H1 levels in the control and SSV mAb groups at the time of LPS injection and 3, 6, 9, 12, and 24 hr later were measured by ELISA and then normalized to the level measured at the time of LPS injection. Values plotted are means ± standard deviation. ^*^Statistically significant differences (*P* < 0.05). (b) Plasma histone H3 levels in mice. (c) Plasma histone H4 levels in mice. (d) Histone H1 levels in lung tissue in the control and SSV mAb groups at the time of LPS injection and then 3, 6, 9, 12, and 24 hr later were measured by ELISA and then normalized to the level measured at the time of LPS injection. Values plotted are means ± standard deviation. ^*^Statistically significant differences (*P* < 0.05). (e) Histone H3 levels in lung tissue. (f) Histone H4 levels in lung tissue.

Histones H1, H3, and H4 levels in plasma and lung tissue over the course of the experiment were determined by ELISA. Sandwich ELISA was used for the quantification of each histone. Histone H1 level was measured by a protocol described in Materials and Methods. Histones H3 and H4 levels were evaluated by a commercial kit. The concentration of each histone was calculated based on the standard curve and thus the concentration varied from pg/mL to mg/mL between histones (data not shown). To avoid the confusion, the concentration of the individual histone at each time point was calculated as a ratio compared with the time of LPS injection (time 0 hr). The plasma histones H1, H3, and H4 levels were significantly higher in the control group than in the SSV mAb group at certain time points (Figures 3(a), 3(b), and 3(c)) (*P* < 0.05). The significant difference was transient and disappeared 24 hr after LPS injection. Histones H1 and H3 levels in the lung tissues were higher in the control group than in the SSV mAb group during the observation time (Figures 3(d) and 3(e)) (*P* < 0.05). Histone H1 levels in the plasma were immediately induced after LPS injection, whereas histones H3 and H4 levels were induced at a late phase. The same induction pattern was observed in histones H1 and H3 levels in lung tissue except for histone H4. For systemic and lung inflammation, histone H1 may trigger immune responses followed by H3 and H4. Histone H4 may not be a critical factor for lung inflammation because histone H4 levels remained stable in control group (Figure 3(f)). These results implied the suppression of histones triggered immune responses by SSV mAb.

### 3.4. Histopathology of Lung Tissue by Hematoxylin-Eosin Staining

**Figure 4:**
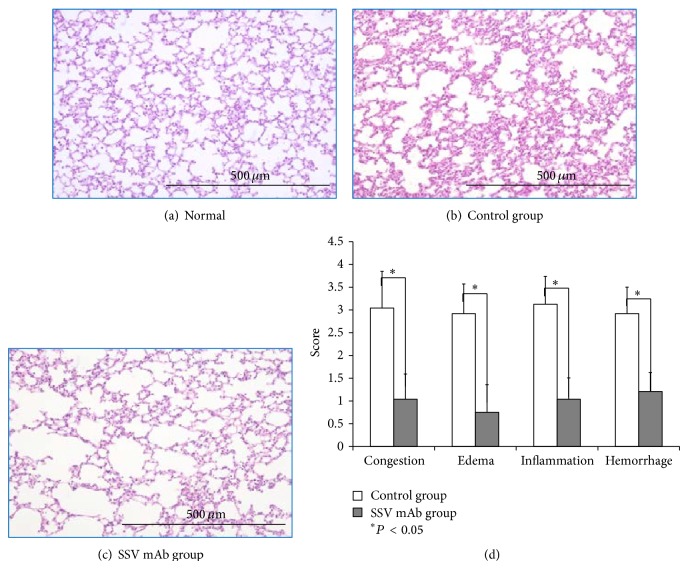
Effect of SSV mAb on histopathology of lung tissue. Lung tissue specimens were obtained 12 hr after LPS injection. The specimens were inflated and fixed in 10% formaldehyde and then subjected to a paraffin blocking procedure. Cross-sections were stained with hematoxylin-eosin and observed under a microscope at 40x magnification. Bars represent 500* μ*m in all panels. (a) A representative sample from a normal mouse that did not receive LPS injection. (b) A representative sample from a mouse that received LPS injection and was treated with a control (anti-mouse IgG1) antibody. (c) A representative sample from a mouse that received LPS injection and was treated with SSV mAb. (d) Images represented by those in panels b and c were divided into 24 separate areas and scored on a 0–4 scale for the level of change (from normal mice) in congestion, edema, inflammation, and hemorrhage. Values presented are means ± standard deviation. ^*^Significant differences (*P* < 0.05).

No histological alterations of the lung tissue were observed in normal mice (Figure 4(a)); however, the interalveolar septa were diffusely edematous and thickened, and inflammatory cell infiltration was observed in the control group (Figure 4(b)). SSV mAb treatment prevented these histological changes (Figure 4(c)). Compared with the control group, the interalveolar septa were thinner and the alveolar surface was greater in SSV mAb-treated mice. Histological scoring of lung injury revealed that all scores in the control group were significantly greater than those in the SSV mAb group (Figure 4(d)).

### 3.5. Plasma Cytokine Levels (TNF-*α*, IL-1*β*, IL-6, and IL-10)

**Figure 5:**
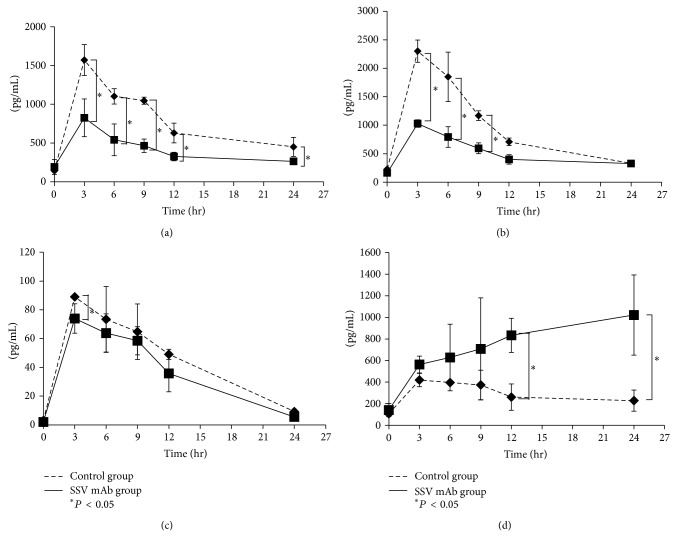
Effect of SSV mAb on plasma cytokine levels. (a) Plasma TNF-*α* levels in the control (dashed line) and SSV mAb (solid line) groups at the time of LPS injection and then 3, 6, 9, 12, and 24 hr later (*n* = 10 per time point) were measured by ELISA. Values plotted are means ± standard deviation. ^*^Statistically significant differences (*P* < 0.05). (b) Plasma IL-1*β* levels in the control (dashed line) or SSV mAb (solid line) groups at the time of LPS injection and then 3, 6, 9, 12, and 24 hr later (*n* = 10 per time point) were measured by ELISA. Values plotted are means ± standard deviation. ^*^Statistically significant differences (*P* < 0.05). (c) Plasma IL-6 levels in the control (dashed line) and SSV mAb (solid line) groups at the time of LPS injection and then 3, 6, 9, 12, and 24 hr later (*n* = 10 per time point). Values plotted are means ± standard deviation. ^*^Statistically significant differences (*P* < 0.05). (d) Plasma IL-10 levels in the control (dashed line) and SSV mAb (solid line) groups at the time of LPS injection and then 3, 6, 9, 12, and 24 hr later (*n* = 10 per time point). Values plotted are means ± standard deviation. ^*^Statistically significant differences (*P* < 0.05).

The plasma TNF-*α* level reached a peak 3 hr after LPS injection in both groups and then began to gradually decrease (Figure 5(a)). Plasma TNF-*α* levels were higher in the control group than in the SSV mAb group during the entire observation time, and the difference reached a statistical significance from 3 to 24 hr after LPS injection (*P* < 0.05). Plasma IL-1*β* levels also reached a peak 3 hr after LPS injection in both groups and then began to gradually decrease (Figure 5(b)). Plasma IL-1*β* levels were higher in the control group during the entire observation time, and the difference reached a statistical significance from 3 to 9 hr after LPS injection (*P* < 0.05). Plasma IL-6 levels also reached a peak 3 hr after LPS injection in both groups and then began to gradually decrease (Figure 5(c)). Plasma IL-6 levels were higher in the control group and the difference reached a statistical significance at 3 h after LPS injection (*P* < 0.05). In contrast to the pattern displayed by histone H1 and the other cytokines, we found that plasma IL-10 levels in the SSV mAb group gradually increased (Figure 5(d)). Plasma IL-10 levels in the control group, however, displayed the typical pattern, reaching a peak 3 hr after LPS injection and then decreasing gradually. Plasma IL-10 levels were higher in the SSV mAb group than in the control group during the entire observation time, and the difference reached a statistical significance 12 and 24 hr after LPS injection (*P* < 0.05).

## 4. Discussion

It has been reported that histones, mainly H3 and H4, released extracellularly in response to inflammatory processes are involved in sepsis-induced endothelial dysfunction, organ failure, and death. Antibodies against H4 protected the mice in sepsis, indicating that H4 is a major mediator of injury in sepsis. Administration of different histone to EA. Hy926 (endothelial cells) demonstrated damage to cells, showing the highest cytotoxicity of histones H3 and H4, whereas histone H1 shows a lower PI fluorescence intensity [1]. Therefore, we hypothesized that it may be important to control histones during LPS-induced inflammatory responses. In this study, SSV mAb was able to suppress histone H1 levels in the plasma and lung tissue. In addition, the levels of histones H3 and H4 were also suppressed. Histone H1 significantly inhibited the binding of SSV mAb to SSV, as demonstrated by competitive ELISAs. Histones H3 and H4 also showed some inhibitory effects, although not significantly, on the binding of SSV mAb to SSV (Figure 1(c)). We speculate that SSV mAb not only binds to histone H1 but also exhibits cross-reactivity against histones H3 and H4 in the plasma. When histones were released extracellularly due to inflammatory processes, the suppression of histone H1 as well as histones H3 and H4 by SSV mAb may have contributed to the prolonged survival of mice injected with a lethal dosage of LPS. Our study also indicated that histone H1 may function as an inflammatory mediator, in addition to its defensive role as a component of NETs. Inflammation also represents an important cause of allograft tissue lesions during ongoing rejection episodes. Adverse graft inflammation creates resistance to the induction of allograft tolerance. The present results further support the previously reported role that histone H1 antibodies play in immunosuppression [9, 10, 18].

TNF-*α* is one of the most important mediators of endotoxic shock, and it causes pathological conditions such as septic shock by decreasing systemic vascular resistance, increasing cardiac output, and directly reducing the force of cardiac contraction [19–23]. In addition, there have been reports that IL-1*β* is also involved in septic shock hemodynamics [24]. IL-6 is an important mediator of the acute phase response and fever and is secreted by macrophages in response to specific microbial molecules known as pathogen-associated molecular patterns [25–29]. Inflammatory cytokines such as TNF-*α*, IL-1*β*, and IL-6 exacerbate a pathological condition by activating clotting mechanisms, which occur via the adhesion of neutrophils to endothelial cells, producing a small thrombus [30]. Here, expression of TNF-*α*, IL-1*β*, and IL-6 was suppressed in the SSV mAb-treated group. In contrast, anti-inflammatory cytokines, such as IL-10, have a role as the immune response control tower. IL-10 is involved in the production of T helper (Th) 1 cells, Th 2 cells, or natural killer T cells [31]. IL-10 has the ability to downregulate the expression of Th1, major histocompatibility complex class II molecules, and costimulatory molecules on macrophages [31, 32]. These sequences of events are involved in the regulation of the Janus kinase/signal transducers and activators of the transcription signaling pathway [33, 34]. We reported in this study that SSV mAb treatment suppressed histone H1 as well as H3 and H4 levels and TNF-*α*, IL-1*β*, and IL-6 expression and increased IL-10 expression in mice injected with LPS, reinforcing the suggestion that the SSV mAb may be clinically useful in inflammation.

We demonstrated the potential of SSV mAb in LPS-induced inflammatory responses via suppression of plasma and lung levels of histones H1, H3, and H4. The limitation of this study was that we did not investigate the effect of SSV mAb on histones H2A and H2B expression. The timing of administration and optimum concentration of SSV mAb also need to be confirmed for future clinical application. We also demonstrated that SSV mAb prolonged the survival of mice injected with lethal dosage of LPS and suppressed plasma cytokine secretion. LPS is a good model to induce the key points of the systemic inflammatory response observed in sepsis, but it did not induce the innate immune response against infection. Further experiments are required to confirm the precise mechanism and effect of SSV mAb on histones in other septic models, such as the cecal ligation and puncture (CLP) model. Our study shows that SSV mAb prolonged the survival of mice injected with LPS and suppressed plasma cytokine secretion. SSV mAb also showed anti-inflammatory and organ-protective effects against acute lung injury. These results suggest that it is important to control histone H1 as well as H3 and H4 levels during inflammation. Our findings demonstrate the potential of SSV mAb as a candidate for drug development for combating inflammation.

## Figures and Tables

**Table 1:** CDR sequences of SSV mAb.

CDR	SSV mAb
V_H_CDR1	GYNMN
V_H_CDR2	NINPYYGSTSYNQKFKG
V_H_CDR3	SPYYSNYWRYFDY
V_L_CDR1	RASSSVSYMH
V_L_CDR2	ATSNLAS
V_L_CDR3	QQWSSNPWT
